# CellTarget: a convex optimisation approach to discover cellular objectives

**DOI:** 10.1038/s41540-026-00700-8

**Published:** 2026-04-03

**Authors:** Mariana Monteiro, James Morrissey, Cleo Kontoravdi

**Affiliations:** 1 The Sargent Centre for Process Systems Engineering, Department of Chemical Engineering, Imperial College London, London, UK; 2 Department of Chemical and Biomolecular Engineering, Johns Hopkins University, Baltimore, MD USA

**Keywords:** Biological techniques, Cell biology, Computational biology and bioinformatics, Systems biology

## Abstract

Metabolic network models are widely used in systems and synthetic biology to study cellular metabolism from microbes to mammals. However, their application to mammalian cells remains limited by the scarcity of intracellular flux measurements relative to network size. As a result, mammalian metabolic models are typically underdetermined and require optimisation-based assumptions to be solved. Unlike microbial systems, mammalian cells do not generally operate under growth-maximising objectives, making objective specification particularly challenging. To address this limitation, we propose CellTarget, an algorithm for inferring cellular objectives directly from experimental flux data. CellTarget combines a convex Flux Balance Analysis (FBA) module with an error-minimisation module that iteratively adjusts objective coefficients to improve agreement between predicted and measured fluxes. Gradients are obtained via implicit differentiation of the convex FBA optimisation problem, enabling backpropagation of the inferred objective coefficients and joint optimisation of both modules. CellTarget is evaluated across producer and non-producer Chinese Hamster Ovary (CHO) cell lines, growth phases, objective dimensionalities and both reduced and genome-scale networks. Across all settings, CellTarget yields accurate forward flux predictions and outperforms a benchmark inverse FBA method. The results reveal substantial non-identifiability of cellular objectives, with predictive accuracy driven more strongly by network constraints than by objective specificity.

## Introduction

Constraint-based metabolic models are commonly solved through optimisation. In the widely used Flux Balance Analysis (FBA) framework^1^, an objective encoded by a coefficient vector is maximised subject to steady-state mass balance constraints. This involves assuming a cellular objective, most commonly growth maximisation^2^. While this assumption is often appropriate for rapidly growing microorganisms, mammalian cells do not appear to prioritise growth under all conditions. Indeed,^3^ reports systematic overprediction of growth rate across multiple conditions and models. This discrepancy reflects the influence of additional metabolic and regulatory processes, meaning that FBA predictions may represent theoretical optima rather than realised cellular behaviour. Alternative objectives have therefore been explored, including optimisation of ATP yield^4^ or biomass production under additional energetic constraints^5^.

Several extensions of FBA attempt to mitigate these limitations. Methods such as Flux Variability Analysis (FVA) characterise the feasible flux space by independently maximising and minimising each reaction^6^. Other formulations incorporate additional biological assumptions, including parsimonious FBA, which minimises total flux while sustaining maximal growth^7^, as well as constraints based on carbon availability, enzyme capacity, or thermodynamic feasibility^8–10^. Alternatively, flux sampling explores the feasible space without imposing an objective by drawing representative flux vectors satisfying the same steady-state and bound constraints^11^. However, for genome-scale networks the resulting high-dimensional solution space can be difficult to interpret for biological applications, motivating approaches that infer cellular objectives directly from experimental data^12^.

One strategy is to consider the inverse of the FBA problem, which seeks to determine a coefficient vector whose optimal solution reproduces experimentally observed fluxes while remaining optimal within the same feasible space defined by stoichiometric constraints and flux bounds. Early inverse FBA approaches, including ObjFind^13^ and BOSS^14^, reformulated the bilevel problem into a single-level non-linear optimisation problem. While these methods identified key metabolic drivers in *E. coli*, they are sensitive to local optima and typically require either predefined objectives or full flux observability. Alternative hypothesis-driven strategies evaluated candidate objectives directly:^15^ systematically compared common objectives across conditions, while^16^ proposed a Bayesian framework for selecting the most plausible objective. Similarly,^17^ introduced a multi-objective formulation in which cellular behaviour is represented as a weighted combination of predefined objectives. These approaches remain limited to testing discrete sets of hypothesised biological goals.

Later formulations incorporated thermodynamic constraints^18^. Explicit integration of Gibbs free energy improves biological plausibility, but the associated computational burden and extensive data requirements restrict these approaches to reduced-scale models. More recent work such as invFBA^19^ uses linear programming duality to obtain polynomial-time tractability for genome-scale networks, yet still requires complete flux measurements. More recently,^20^ proposed a Bayesian optimisation framework for general inverse problems, with one case study involving in silico metabolic objective inference.

A recurring limitation among most approaches is the lack of backwards-forward compatibility, that is, the inability to recover the original experimental fluxes when re-solving the FBA problem with the inferred objective. This is increasingly difficult under partial observability conditions and stems from the fundamental property of underdetermination in FBA: multiple flux distributions may yield the same optimal objective value, rendering the mapping from objective to flux non-injective. Consequently, even when a method identifies an objective consistent with the data, the fluxes reconstructed via forward FBA may not match the measurements.

In light of these limitations, there remains a need for an inverse FBA framework that can operate under partial flux observability, scale to large metabolic networks and maintain backwards–forward compatibility. CellTarget addresses this gap by formulating the problem as a differentiable bilevel optimisation, in which gradients are obtained by implicitly differentiating the solution of the lower-level convex FBA problem with respect to the objective coefficients. These gradients enable refinement of the objective function to improve agreement between forward FBA predictions and experimental fluxes.

CellTarget learns the optimal weights (i.e. cellular objectives) by minimising the difference between the predicted and experimental fluxes, while ensuring stoichiometric feasibility and bound constraints are satisfied at all times. The formulation and implementation of CellTarget is presented herein along with its evaluation in both synthetic and experimental case studies.

## Results

### CellTarget identifies objectives across convex basis functions under full observability

**Fig. 1:**
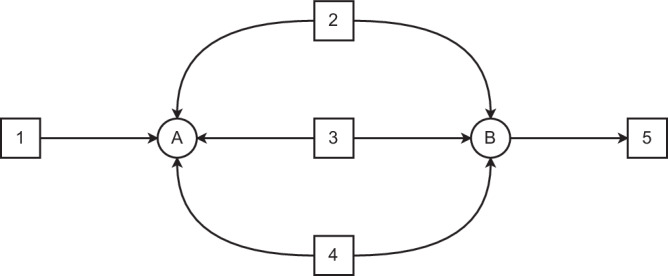
Network model proposed in ref. ^21^. The schematic illustrates the stoichiometric structure of the metabolic network, including metabolites, reactions and their interconnections.

CellTarget is first applied to a case study of a reduced-size network taken from ref. ^21^, where full observability is assumed. The network model is shown in Fig. 1 and the bounds and stoichiometry of the network can be found in Tables 1 and 2. Full Observability means that all fluxes are assumed to be measured. The measured fluxes (referred to as Ground Truth in Table 1) represent the predicted fluxes obtained using the bounds and the stoichiometric constraints, by solving an optimisation problem that minimises the Euclidean norm of the flux vector. These values serve as the reference data for the CellTarget inference exercise.

**Table 1:** Flux bounds for each reaction in the metabolic network model

Flux Index	Lower Bound	Upper Bound	In silico data
**v**_1_	−1	1	4.33 × 10^−6^
**v**_2_	−10,000	−2	−2
**v**_3_	0	2	1
**v**_4_	−2	10,000	1
**v**_5_	0	10,000	4.33 × 10^−6^

*In silico* data represents the predicted fluxes obtained using these bounds and the stoichiometric constraints, and serve as the reference data for the CellTarget inference exercise.

**Table 2:** Stoichiometric matrix *S* of the metabolic network

Metabolite	*v*_1_	*v*_2_	*v*_3_	*v*_4_	*v*_5_
*M*_1_	1	−1	−1	−1	0
*M*_2_	0	1	1	1	−1

**Fig. 2:**
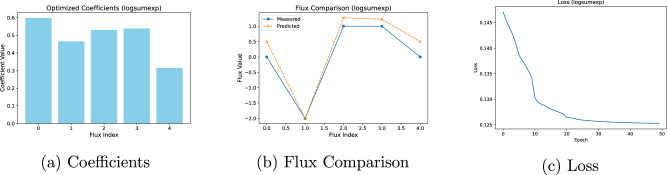
Analysis and evaluation of the log-sum-exp basis function. **a** Inferred objective coefficients. **b** Comparison between predicted and observed flux values. **c** Loss function trajectory during training.

**Fig. 3:**
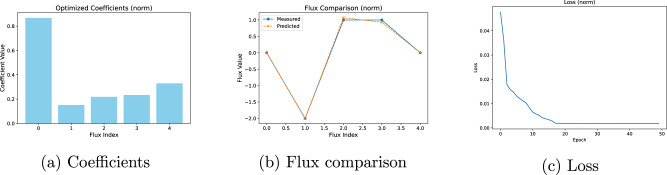
Analysis and evaluation of the norm basis function. **a** Inferred objective coefficients. **b** Comparison between predicted and observed flux values. **c** Loss function trajectory during training.

**Fig. 4:**
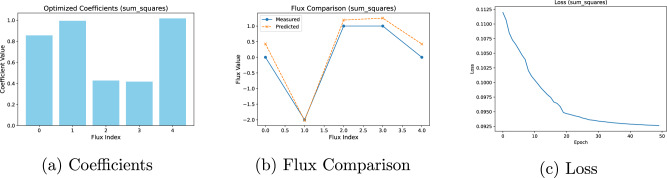
Analysis and evaluation of the sum-of-squares basis function. **a** Inferred objective coefficients. **b** Comparison between predicted and observed flux values. **c** Loss function trajectory during training.

Figures 2, 3 and 4 present the results of applying CellTarget with three different convex basis functions. Convex basis functions are used here to provide a well-behaved optimisation landscape for estimating the objective coefficients. In all cases, the optimisation yields non-zero coefficients. For two of these functions (sum_squares and norm), the coefficients are concentrated on a subset of fluxes, suggesting sparsity in the inferred objective. The exception is logsumexp, which produces a more uniform coefficient distribution, indicating a different optimisation behaviour. Notably, norm also exhibits the best backwards-forwards compatibility, meaning that coefficients inferred from the backwards (cellular objective inference) step better reproduce the same fluxes when re-applied in the forward prediction step. Although the coefficient profiles differ, even in this small network (which contains a cycle), multiple coefficient configurations achieve the objective equally well. These differences likely arise from the intrinsic properties of the convex basis functions, some encouraging sparsity (sum_squares) others promoting uniformity (norm).

Despite variations in coefficients, the predicted fluxes align closely with the experimental measurements in all three cases, demonstrating good recovery of the true objectives under full observability. This reinforces two points: (i) multiple coefficient profiles can yield equally accurate flux predictions, and (ii) for problems of this size, CellTarget’s results are more strongly determined by stoichiometry and flux bounds than by the specific form of the convex basis function. The loss curves for all three functions show rapid convergence within the first few epochs, followed by flattening.

To understand whether the obtained coefficients are local minima, the Karush-Kuhn-Tucker (KKT) conditions were calculated for the different solutions^22,23^. This verification ensures that the solutions meet the theoretical requirements for optimality. Implementation details can be found in the Supplementary Information Section ‘Calculation of KKT conditions for the Full Observability Case’ and the results of this exercise can be found on Table 7 in that same Section. All of the convex basis functions satisfy the primal feasibility condition *S*.*v* = 0 to machine precision (ranging between 10^−10^ to 10^−13^). With regards to the bounds feasibility, in all cases, the predicted fluxes are within the bounds as the conditions are = 1 for each case. The stationary residuals range from 10^−5^ to 10^−7^. All cases satisfy the complementary slackness as well, as the lower and upper bounds range between 10^−6^ and 10^−11^. Overall, all three formulations achieve solutions that abide by KKT conditions, with norm exhibiting the tightest residuals and logsumexp showing slightly larger (yet small) stationarity and complementarity gaps. As noted earlier in this Section, the ground-truth in silico data were produced under a norm objective, which likely explains why the norm formulation attains smaller stationarity and complementarity residuals. Alternative convex basis functions (sum_squares, logsumexp) impose different geometry, which can explain the slightly larger observed residuals.

#### CellTarget recovers objectives that reconcile forward and backwards flux predictions

**Fig. 5:**
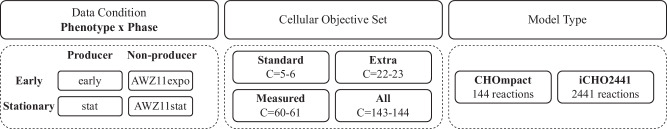
Overview of the study design. CellTarget is evaluated across four experimental conditions defined by cellular phenotype and growth phase, in combination with multiple cellular objective sets of increasing dimensionality and two metabolic model resolutions.

CellTarget is assessed systematically across a range of cell culture conditons and model types, representing various sources of variablilty in model findings, as summarised in Fig. 5. Four experimental conditions are considered under the Data Condition axis, defined by the combination of cellular phenotype (producer or non-producer CHO cell line) and growth phase (exponential or stationary). Experimental data are drawn from^24^ and^25^.

The effect of cellular objective dimensionality is investigated using four candidate objective sets. The ‘Standard’ set comprises a reduced, biologically motivated subset of reactions (growth, monoclonal antibody (mAb), lactate, glucose, asparagine and glutamine). The ‘Extra’ set includes all measured extracellular fluxes, while the ‘Measured’ set considers all experimentally measured fluxes, including intracellular. Finally, the ‘All’ configuration treats the full reaction space of the model as potential cellular objectives. For the non-producer cell line, the mAb synthesis reaction is excluded from the candidate objective set.

Sensitivity to model size is assessed by performing the analysis on both a reduced metabolic network (CHOmpact with 144 reactions^26^) and the genome-scale iCHO2441 model^3^.

#### Effect of phenotype and cell culture phase

**Fig. 6:**
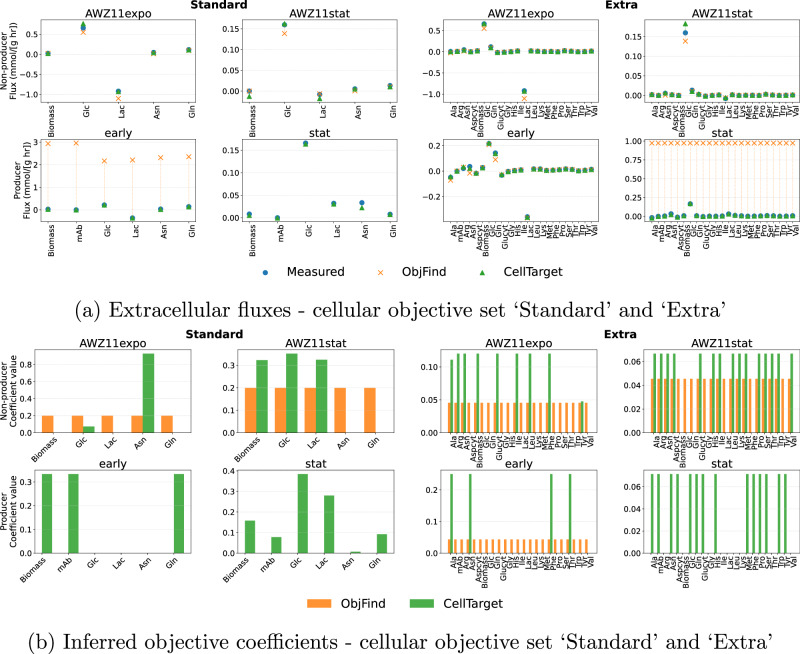
Benchmarking CellTarget against ObjFind* across different cell culture phases and phenotypes using CHOmpact. Comparison of experimental and predicted extracellular fluxes from CellTarget and ObjFind* (**a**) and inferred objective coefficients (**b**). Missing values for ObjFind* correspond to failed optimisation runs.

Figure 6 presents the coefficients (inferred cellular objectives) and the corresponding backwards–forward compatibility results for both cell lines, for all cell culture phases. In each case, the experimental fluxes are compared with forward FBA predictions obtained using the objectives inferred by CellTarget and ObjFind* (benchmark algorithm; see ‘Methods’ Section)

The fluxes predicted by CellTarget consistently match experimental values across all conditions considered. In contrast, ObjFind* appears more sensitive: in some cases it tracks the data well (e.g. in the non-producer cell line), while in others, most notably in producer cell line conditions, it fails to do so. ObjFind* tends to assign coefficients of the same magnitude to all considered cellular objectives. This cannot solely be a consequence of the constraint that coefficients must sum to one, since this is imposed in both methods. CellTarget is better able to identify distinct objective structures across different phenotypes and cell culture phases, reflecting condition-dependent cellular priorities, especially for a smaller set of candidate cellular objectives. For the non-producer cell line, the cellular coefficients obtained with CellTarget and ObjFind* result in good agreement between predicted and experimental fluxes. Nonetheless, ObjFind* assigns the same weight to all considered cellular objectives. This is in agreement with some of the good results parsimonious FBA has reported^27^. However, such uniform objectives are less informative in the present context, as they do not reveal prioritisation or trade-offs among competing cellular functions.

For the ‘Standard’ set of cellular objectives, in the exponential phase of the producer cell line, higher weights are assigned to biomass and mAb formation, whereas in the non-producer cell line a higher weight is given to asparagine. In stationary phase, higher weights are assigned to glucose uptake, lactate secretion and biomass for both cell lines, consistent with a shift from growth-driven objectives towards maintenance-related metabolic demands.

For the ‘Extra’ set of cellular objectives, CellTarget assigns approximately uniform non-zero weights across many reactions, particularly amino acid exchange fluxes. This behaviour most likely reflects non-identifiability of the objectives under the available data, rather than meaningful biological prioritisation.

Supplementary Fig. 1 (in Supplementary Information, Section ‘Loss Plots’) shows the loss history across training epochs for CellTarget. The trajectories reflect local convergence following the multi-start initialisation routine, which provides an initial exploration of the parameter space. More details about the initialisation routine can be found in the Supplementary Information Section ‘Initialisation’. Each colour corresponds to the result of a parallel initialisation. In all four subplots, the loss curves flatten after the first few epochs, indicating that most parameter adjustments occur almost immediately and then stabilise. These profiles suggest that the optimiser is already operating in a relatively flat, satisfactory region of the loss surface, where further improvements are limited. Importantly, even in runs with little visible loss reduction after the first step, forward–backwards compatibility remains satisfactory, suggesting that the optimiser’s limited activity may also reflect a lack of necessity for major adjustments rather than a failure to improve.

#### Effect of cellular objective dimensionality

**Fig. 7:**
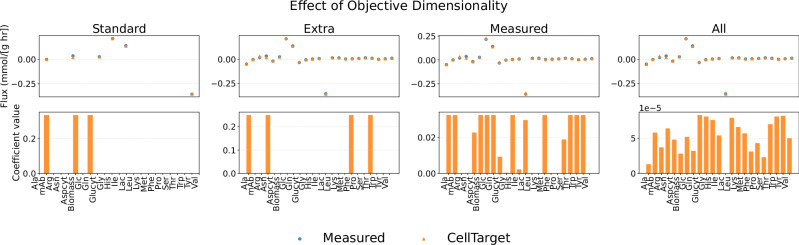
Effect of cellular objective dimensionality in CellTarget. Comparison of experimental and CellTarget-predicted extracellular fluxes (top row) and inferred objective coefficients (bottom row) for increasing objective dimensionality (standard, extra, measured, all) in early-phase CHO cell cultures.

Figure 7 depicts the effect of increasing the dimension of the set of cellular objectives considered. As previously explained in Fig. 5, four sets of cellular objectives were studied: biologically motivated subset (‘Standard’), to extracellular fluxes, measured fluxes and the full reaction space.

Increasing the number of candidate cellular objectives does not affect the agreement between predicted and experimental fluxes. This suggests that expanding the set of cellular objectives might not always yield additional fitting benefit.

Increasing the dimensionality instead redistributes the inferred objective coefficients. This indicates that multiple cellular objectives are equally compatible with the observed fluxes, highlighting the non-identifiability of a unique ‘true’ cellular objective from the available data. As the objective space expands, coefficient values become more broadly distributed. In contrast, lower-dimensional objective sets allow CellTarget to concentrate weight on a reduced number of objectives. Despite forcing the sum of cellular objectives to be equal to 1, increasing dimensionality still relaxes this constraint, decomposing the objective across multiple metabolites.

The lack of convergence toward a unique coefficient structure further suggests that cellular behaviour cannot be explained by optimisation of a single dominant objective. Moreover, the flux constraints appear to play a more decisive role than the objective itself, as the inferred objectives remain flexible within the feasible flux space. One example of this is observed in some stationary phase experiments, using the ‘all’ objective set (see Supplementary Figs. 4 and 6), where the largest inferred coefficients correspond to reactions F104 and F144, associated with ATP demand.

Supplementary Table 1 (in Supplementary Information, Computational Effort Section) shows the CPU and Walltime usages for the different cellular objective dimensionality conditions. CPU time corresponds to the cumulative processor time summed across all allocated five cores, whereas walltime denotes the elapsed real time to completion. The computational cost does not scale monotonically with the increased size of cellular objectives. This observation is consistent with the notion that increasing the cellular objective space may facilitate parameterisation by relaxing the optimisation landscape rather than strictly increasing its complexity.

#### Effect of model size

**Fig. 8:**
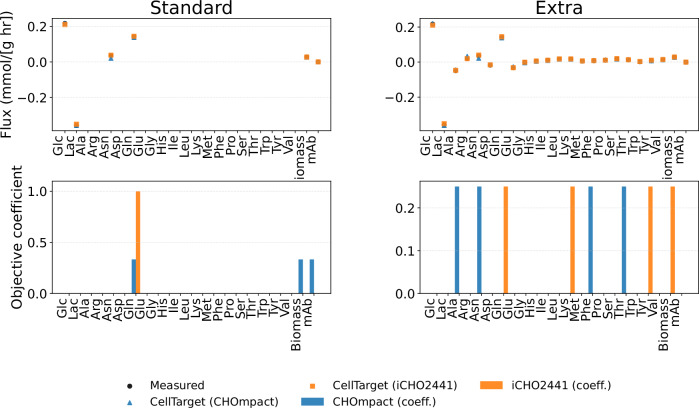
Effect of model resolution for producer cell line in exponential phase. This effect is evaluated across two cellular objective dimensionalities (‘Standard’ and ‘Extra’).

Figure 8 depicts the effect of model size (CHOmpact vs iCHO2441) for the producer cell line in the exponential phase case with two types of cellular objectives (‘Standard’ and ‘Extra’). The agreement between predicted and experimental fluxes is preserved across both model sizes. This indicates that increasing the degrees of freedom of the model does not yield additional gains in predictive accuracy, particularly given that the baseline agreement is already high.

Despite comparable flux predictions, the inferred cellular objectives differ between model sizes for the same experimental data. This highlights a lack of identifiability of the cellular objective that persists across network scales. In the ‘Standard’ case (left panel), CHOmpact assigns higher weights to biomass growth, mAb synthesis and glutamine uptake, while iCHO2441 assigns a large weight to glutamine alone. In the ‘Extra’ case (right panel), CHOmpact assigns uniform importance to the exchange rates of amino acids (threonine, alanine, phenylalanine and asparagine), while iCHO2441 to glutamine, biomass growth and tyrosine and lysine exchange rates. iCHO2441 consistently identifies glutamine uptake as an important cellular objective. Since CHOmpact lumps several intracellular reactions, CellTarget is forced to combine multiple exchange fluxes to approximate the observed experimental data.

In Supplementary Information, Effect of Model Size Section, the effect of model size is shown for each cell line and growth phase across all cellular objective sets. When either the metabolic network or the size of the cellular objective set is small, larger coefficients tend to appear. Importantly, this reflects a scaling effect rather than a change in the underlying cellular priorities, as coefficients are normalised over fewer competing objectives.

In the producer cell line, in exponential phase (see Supplementary Figs. 5 and 9), glucose uptake and biomass growth objectives are observed in both model sizes. In stationary phase (see Supplementary Figs. 6 and 10), glucose uptake, glucose uptake, lactate and acetate secretion and biomass/protein synthesis are reactions common across model types. In the non-producer cell line, in exponential phase (see Supplementary Figs. 3 and 7), asparagine and glutamine appear as the top contendors for cellular objectives. While in stationary phase (see Supplementary Figs. 4 and 8), exchange reactions are also predominant (glucose, lactate and asparagine), but some intracellular reactions relating urea and arginine also appear with a larger coefficient.

In most of the ‘Standard’ cases, iCHO2441 selects a single dominant cellular coefficient, either glutamine, glucose or asparagine uptake rates. These nutrients are known to be highly correlated with CHO cell growth. CHOmpact, however, often shows a combination of multiple objectives alongside biomass. To a lesser extent, this effect is also observed in the ‘Extra’ cases. iCHO2441 is generally sparser, showing fewer, higher-weighted cellular objectives while CHOmpact often relies on a broader combination of exchange fluxes to satisfy the observed phenotypic constraints. This likely reflects a compensation mechanism for the topological lumping in the reduced model.

In contrast to objective dimensionality, model size has a pronounced impact on computational cost. Supplementary Table 2 (in Supplementary Information ‘Computational Effort Section’) shows the CPU and Walltime usage for CHOmpact and GEM models under standard and extracelullar cellular objective configurations. Using iCHO2441 is approximately an order of magnitude more expensive than using CHOmpact, and the size of the cellular objective has practically no impact. In fact, for iCHO2441, using a smaller set of cellular objectives (‘Standard’) takes longer to run. This corroborates what was observed in the previous Section; the size of the network and subsequently the constraint dimensionality have a larger impact on computational time, rather the number of cellular objectives considered. Increasing the cellular objective dimensionality effectively introduces slack, which can facilitate the optimisation.

## Discussion

Biological systems are known to exhibit degeneracy, a feature that grants them robustness to perturbations by enabling multiple solutions to achieve similar functional outcomes^28^. This inherent flexibility makes it challenging to mathematically define a single, consistent cellular objective, even when the system is simplified. In this work, CellTarget is introduced: an algorithm designed to infer cellular objectives that explain observed flux distributions, by learning those objectives directly from experimental fluxes. This is achieved through convexification techniques that approximate the inherently non-convex space defined by FBA.

A preliminary case study under full observability demonstrates CellTarget’s ability to recover objective functions that closely reproduce experimental fluxes. While different convex basis functions yield distinct coefficient profiles, the predictive performance remains consistently high. This indicates that multiple objective structures can lead to similar forward behaviour, an expected consequence of the degeneracy of the solution space. This further underscores the limited identifiability of the objective parameters, making it difficult to resolve a unique biological ‘truth’ without additional information. KKT analysis confirms that the obtained solutions can be considered local optima. Notably, these minima were achieved without any pre-initialisation of the cellular objectives; all coefficients were instead randomly assigned values between 0 and 1, demonstrating the method’s ability to converge to optima from uninformed starting points, in problems of this size.

In our systematic assessment across differing phenotypes and growth phases, CellTarget consistently outperforms ObjFind* in forward predictive accuracy, supporting its effectiveness under both full and partial observability. The learned objectives frequently span multiple fluxes, suggesting that cells may follow multi-objective strategies rather than optimising a single goal. The observed loss trajectories further support the presence of local minima and flat regions in the loss surface, highlighting the importance of both convex basis function choice and the multi-start initialisation strategy.

Regarding the loss evolution across the case study under partial observability, the optimisation step primarily serves to certify that the initial solution is at least locally optimal. While the initialisation yields strong starting points in the presented scenarios, often resulting in minimal further improvement during training, this favourable behaviour cannot be assumed in general. In more complex or noisier datasets, or with different convex basis functions, the initialisation alone may fall short. In such cases, the optimiser plays a critical role in fine-tuning the coefficients, adapting to experimental variability and recovering meaningful cellular objectives.

The study of the impact of cellular objective dimensionality revealed that increasing the size of the candidate objective set does not improve predictive accuracy. Instead, objective expansion primarily redistributes weight across additional degrees of freedom, revealing substantial redundancy in how cellular behaviour can be represented. The non-monotonic scaling of computational cost with objective dimensionality suggests that larger objective spaces do not necessarily increase optimisation difficulty. In some cases, expanding the objective set appears to relax the optimisation landscape, facilitating parameterisation rather than complicating it. When expanding the dimension of cellular objective, the limiting factor appears to be the availability of data capable of resolving metabolic priorities beyond a low-dimensional set.

Building on this, the comparison between CHOmpact and iCHO2441 reveals a clear scalability trade-off. While genome-scale resolution substantially increases computational cost, it does not yield corresponding improvements nor deterioration in predictive accuracy. However, it appears to improve the identifiability of the metabolic drivers. In the majority of cases, iCHO2441 model isolates one or two dominant cellular objectives, while CHOmpact frequently assigns distributed weights across a larger set of variables. This suggests the reduced model compensates for topological lumping by distributing objective weights across multiple fluxes. Taken together, these findings suggest that neither increasing objective dimensionality nor expanding model scale guarantees better identifiability.

An important consideration of this method is the impact of experimental error. This is accounted for in the definition of the flux bounds at the lower level of CellTarget. Smaller measurement errors result in tighter flux bounds which impact the feasible space of the FBA problem. This overall improves the cellular objective identifiability because fewer sets of objectives can explain the data. Similarly to standard FBA, overly constrained flux bounds might affect the feasibility of the problem and can make the numerical convergence of the optimisation harder. This poses the broader question of which data would be needed to better characterise true cellular objectives. The degeneracy of the problem could be reduced if diverse data were to be included. For instance, transcriptomic or proteomic data could introduce soft constraints on pathway activity, discouraging solutions that rely on highly redundant pathways. Likewise, thermodynamic information could further reduce non-identifiability by restricting the feasibility of specific reactions. Integrating such heterogeneous information would likely necessitate a methodological framework explicitly designed to accommodate multiple data sources and dimensions.

The assignment of non-zero coefficients to specific metabolites indicates that including those fluxes in the cellular objective contributes to minimising the prediction error between model outputs and experimental data. These inferred objectives should therefore be interpreted as the optimiser identifying fluxes whose inclusion helps improve the fit. Crucially, this identification is shaped by the parameterisation of the loss function. Improvement may arise from different mechanisms: for instance, high-magnitude fluxes can dominate the loss and therefore require stronger alignment; alternatively, fluxes with initially large deviations from experimental values may benefit from direct optimisation pressure. Moreover, due to stoichiometric coupling within the network, emphasising a particular flux in the objective may indirectly enhance the accuracy of other flux predictions. As such, the resulting weights reflect not only the direct predictive value of each flux, but also its structural influence on the overall solution space. The optimisation landscape is also characterised by stiffness, with certain parameters exhibiting high sensitivity to small changes in the data or initialisation, while others remain largely insensitive–complicating gradient-based optimisation and contributing to the prevalence of flat regions. Still, it is worth noting that the conceptual bilevel problem structure was preserved; the observed behaviours and limitations therefore reflect the intrinsic properties of the underlying formulation.

Importantly, CellTarget aligns with both Type 1 (goal-driven) and Type 2 (hypothesis-driven) objective function categories, as proposed by ^12^. Under the Type 1 paradigm, CellTarget can be configured to simulate predefined biological or engineering goals–such as maximising productivity–while under the Type 2 paradigm, it can infer plausible cellular objectives from data, supporting the generation of mechanistic hypotheses. This flexibility broadens its applicability across both design-oriented and discovery-driven modelling efforts.

As emphasised by several authors^12,13,19^, the objective function in constraint-based modelling should not be mistaken for the literal biological goal of the organism–it is, fundamentally, a modelling assumption. In the context of CellTarget, the inferred objective reflects directions that reduce loss under given constraints. These directions may encode meaningful biological priorities, but they must always be interpreted with care and in light of the modelling context.

An important limitation is the absence of thermodynamic constraints in the present formulation; incorporating Gibbs free energy estimates in future iterations could reduce the feasible space and improve both interpretability and identifiability of the inferred objectives. Future work could also investigate the integration of transcriptomic, proteomic, or regulatory circuit data to further constrain or interpret the inferred objective functions, providing a more comprehensive view of cellular priorities and control strategies.

## Methods: CellTarget

**Fig. 9:**
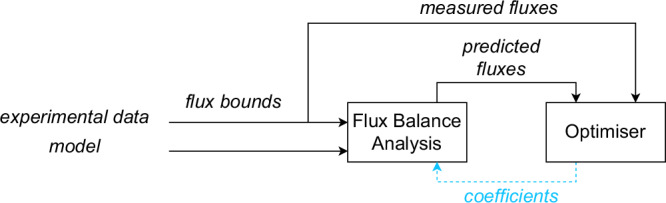
Bilevel structure of CellTarget. The optimiser iteratively updates objective coefficients to align FBA-predicted fluxes with experimental data. Experimental data is used differently in each module: the FBA module receives flux bounds derived from experimental measurements, whereas the optimiser receives the median of the measured flux values for comparison with predicted fluxes.

To address the limitations of previous inverse FBA approaches, particularly the lack of forward–backwards compatibility (i.e. the inability to recover similar fluxes when running the inferred objectives in the forward direction), reliance on predefined objectives and limited scalability, this manuscript proposes CellTarget, a differentiable bilevel optimisation framework for inferring metabolic objectives from flux data. Figure 9 shows a diagram of CellTarget.

**Fig. 10:**
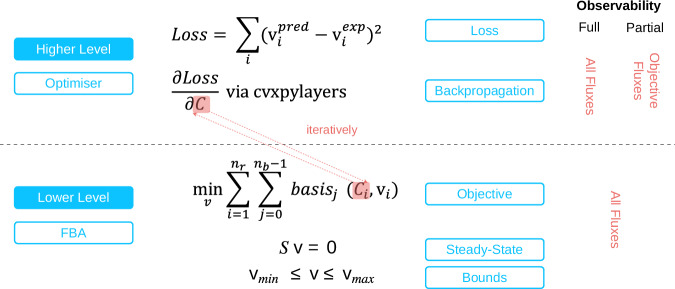
Hierarchical optimisation structure under full and partial observability conditions. In the full observability case, the upper-level optimiser has access to all fluxes, whereas in the partial observability case, the upper-level optimiser only has access to selected fluxes–typically those that can be reliably measured–and uses them to compute a loss function based on the discrepancy between predicted and experimental values. This partial observability does not require that all measured fluxes be included in the higher-level loss; only a chosen subset may be used - and, in fact, only a subset of candidate objectives fluxes may be used in the case studies shown in this manuscript.

Unlike methods that assume the objective lies within a predefined set or that require full flux enumeration, CellTarget defines a flexible class of convex surrogate functions parameterised by a vector *C*, and leverages automatic differentiation through a differentiable convex optimisation layer implemented in CVXPY to enable gradient-based objective inference^29^. The overall algorithm and inter-layer communication are illustrated in Fig. 10. CellTarget estimates the optimal weights *C* (i.e. the cellular objectives) by minimising the discrepancy between predicted and experimental fluxes, while strictly maintaining stoichiometric feasibility and bound constraints. This is accomplished by embedding the inner optimisation problem as a differentiable layer via cvxpylayers. In the full observability case, the higher level uses all measured fluxes, whereas in partial-observability mode, only the available subset is incorporated. The lower-level problem always solves the complete FBA system using the coefficients inferred at the upper level.

Let ${\mathcal{O}}$ denote the index set of candidate objective fluxes (selected according to the configuration standard, extra, measured, or all), and let ${{\bf{v}}}_{{\mathcal{O}}}$ be the corresponding subvector of the flux vector **v**. Given experimental flux data ${{\bf{v}}}_{{\mathcal{O}}}^{\exp }$, the aim is to identify a weighting vector ${\bf{C}}\in {{\mathbb{R}}}^{| {\mathcal{O}}| }$ such that the following lower-level optimisation problem reproduces the observed fluxes on ${\mathcal{O}}$:

1 $${{\bf{v}}}^{* }({\bf{C}})=\arg \,\mathop{\min }\limits_{{\bf{v}}\in {{\mathbb{R}}}^{n}}\,{\mathcal{F}}({\bf{C}},{{\bf{v}}}_{{\mathcal{O}}})$$

subject to

2 $${\bf{S}}{\bf{v}}={\bf{0}}$$

3 $${{\bf{v}}}_{\min }\le {\bf{v}}\le {{\bf{v}}}_{\max }$$

where ${\bf{S}}\in {{\mathbb{R}}}^{m\times n}$ is the stoichiometric matrix, **v** is the full flux vector, and ${\mathcal{F}}({\bf{C}},{{\bf{v}}}_{{\mathcal{O}}})$ is a differentiable surrogate objective. For surrogate objectives that are concave in ${{\bf{v}}}_{{\mathcal{O}}}$, the optimisation is equivalently posed as a maximisation problem.

The strict steady-state mass balance constraint **Sv** = **0** was relaxed to a two-sided tolerance − *ε*≤**Sv**≤*ε*, implemented as **Sv**≤*ε* and − **Sv**≤*ε*. To facilitate numerical convergence, *ε* = 10^−2^ was used for CHOmpact and *ε* = 10^−1^ for iCHO2441. In objectives involving logarithmic or reciprocal terms, a small numerical safeguard *δ* = 10^−6^ was added to ensure well-posedness.

The upper-level problem identifies **C** by minimising a squared-error loss evaluated on the same index set ${\mathcal{O}}$:

4 $$\mathop{\min }\limits_{{\bf{C}}}\,{\mathcal{L}}({\bf{C}})=\frac{1}{| {\mathcal{O}}| }\mathop{\sum }\limits_{i\in {\mathcal{O}}}{({{\bf{v}}}_{i}^{* }({\bf{C}})-{{\bf{v}}}_{i}^{\exp })}^{2}.$$

The coefficient vector was constrained to lie on the probability simplex to prevent trivial rescaling, i.e.

5 $${C}_{i}\ge 0\,\forall i,\,\mathop{\sum }\limits_{i}{C}_{i}=1.$$

This was enforced via a softmax reparameterisation ${\bf{C}}=softmax({\boldsymbol{\theta }})$, ensuring positivity and unit sum throughout optimisation.

Table 3 presents different convex basis formulations that can be considered for the objective function ${\mathcal{F}}({\bf{v}})$ used in inverse FBA.

**Table 3:** Summary of convex basis functions

Mode	Type	Actual Objective
norm	Convex	Minimise ∥**C** ⊙ **v**∥
sum_squares	Convex	Minimise $\sum {({{\bf{C}}}_{i}{{\bf{v}}}_{i})}^{2}$
logsumexp	Convex	Minimise $\log \sum \exp ({{\bf{C}}}_{i}{{\bf{v}}}_{i})$
inverse_log	Concave	Maximise $\log (\sum ({{\bf{C}}}_{i}{{\bf{v}}}_{i}))$

The inverse FBA problem defined in Eq. (1) is a bilevel optimisation problem, where the lower (inner) problem defines a convex programme and the higher (outer) problem seeks to identify the optimal parameter vector **C** that best reproduces measured flux data ${{\bf{v}}}^{\exp }$.

To solve this problem, the fact that the lower problem is convex and differentiable with respect to **C** is leveraged. Specifically, the lower-level optimisation is implemented using CVXPY, which exposes it as a differentiable layer using cvxpylayers^29^. This allows gradients to be propagated through the solution of the lower convex programme during training.

At each iteration, the algorithm performs the following steps:

1. Solve the lower optimisation problem v^*^(**C**) for the current value of **C**.
2. Compute the loss ${\mathcal{L}}({\bf{C}})=MSE({{\bf{v}}}^{* }({\bf{C}}),{{\bf{v}}}^{\exp })$.
3. Backpropagate the loss through the convex optimisation layer to update **C**.

For CellTarget to be applicable, the following conditions must be satisfied:

- The lower problem must be convex in **v** for fixed **C**.
- The surrogate objective ${\mathcal{F}}({\bf{C}},{\bf{v}})$ must be convex in **v** and differentiable with respect to **C**.
- The lower problem must comply with the Disciplined Convex Programming (DCP) ruleset and be Disciplined Parametrised Programming (DPP) to allow symbolic gradients to be propagated through cvxpylayers.
- Partial or full observability of ${{\bf{v}}}^{\exp }$ must be available to evaluate the loss.

This approach avoids the need for explicitly computing KKT conditions or manually deriving gradients, while retaining exact adherence to the convex constraints of the metabolic network model. KKT conditions are first-order necessary and sufficient conditions for a given solution to a nonlinear programming problem to be optimal^22,23^. While these conditions can be derived analytically, doing so becomes cumbersome for large or nested problems. These conditions are explicitly derived for a small toy model, under full observability.

### Initialisation

Given the nature of the inverse FBA problem, the choice of initial guess plays a critical role in guiding the optimisation process, with better initialisations often leading to faster and more reliable convergence^30^. For the difficult cases of partial observability, an initialisation routine was implemented that leverages a surrogate model to explore the parameter space more intelligently and identify promising initialisations that lead to lower loss. The initialisation is a multi-start strategy in which five independent optimisation runs are launched in parallel, each with a different initialisation of the objective coefficient vector *C*. After training, the solution with the lowest final loss is selected.

Let ${\bf{C}}\in {{\mathbb{R}}}^{d}$ denote the coefficient vector parameterising the basis function representation of intracellular fluxes. A loss function *f*(**C**) is defined that evaluates the reconstruction error after a limited number of gradient descent iterations. Specifically, for each candidate **C**, a short optimisation is run as:

6 $$f({\bf{C}})={\mathcal{L}}(\widehat{{\bf{v}}}({\bf{C}}),{{\bf{v}}}^{\exp }),$$

where $\widehat{{\bf{v}}}({\bf{C}})$ is the predicted flux vector obtained after a fixed number of optimisation epochs starting from **C**, and ${{\bf{v}}}^{\exp }$ represents the experimentally measured fluxes. The loss function ${\mathcal{L}}(\cdot ,\cdot )$ to minimise is the Mean Squared Error (MSE) between predicted and experimental fluxes, and was implemented in PyTorch^31^.

7 $${\mathcal{L}}({\bf{c}})={\parallel {{\bf{v}}}_{meas}^{\exp }-{{\bf{v}}}_{meas}({\bf{c}})\parallel }_{2}^{2}$$

The goal is to find an initial **c**_0_ such that:

8 $${{\bf{c}}}_{0}=\arg \mathop{\min }\limits_{{\bf{C}}\in {[0,1]}^{d}}{\mathcal{L}}({\bf{c}})$$

where *d* is the number of measured fluxes and [0, 1]^*d*^ is the search domain for the coefficients. *f*(**C**) is minimised over the bounded domain using Bayesian Optimisation (BO). A Gaussian Process (GP) model is trained on an initial design of Sobol-sampled points, and iteratively refined using an acquisition function. The BO loop is as follows:

1. Generation of a quasi-random set of initial candidates using Sobol Sequence^32^.
2. Each sampled point **c**^(*i*)^ is evaluated by computing the final value of the loss ${\mathcal{L}}({{\bf{c}}}^{(i)})$ after a short local optimisation. Each loss value *y*^(*i*)^ is thus associated with each candidate point.
3. A GP is used as the surrogate model for the black-box function ${\mathcal{L}}({\bf{c}})$. The GP is defined as:
  9 $${\mathcal{L}}({\bf{c}}) \sim {\mathcal{GP}}(\mu ({\bf{c}}),k({\bf{c}},{{\bf{c}}}^{{\boldsymbol{{\prime} }}}))$$
  where *μ*( ⋅ ) is a constant mean function and *k*( ⋅ , ⋅ ) is the radial basis function (RBF) kernel. This was implemented in the GPyTorch interface^33^.
4. The GP hyperparameters are optimised by maximising the marginal log-likelihood, using the LBFGS optimiser^34^:
  10 $$\log p({\bf{y}}| {\bf{X}})=-\frac{1}{2}{{\bf{y}}}^{\top }{(K+{\sigma }^{2}I)}^{-1}{\bf{y}}-\frac{1}{2}\log | K+{\sigma }^{2}I| -\frac{n}{2}\log (2\pi )$$
5. The next candidate point **c**^next^ is selected by maximising the acquisition function. In this case, we use the Lower Confidence Bound (LCB), which is the negative of Upper Confidence Bound (UCB):
  11 $${\mathrm{LCB}}({\bf{C}})=\mu ({\bf{C}})-\beta \cdot \sigma ({\bf{C}}),$$
  where *μ*( ⋅ ) and *σ*( ⋅ ) are the posterior mean and standard deviation from the GP, and *β* > 0 is an exploration parameter. This acquisition function is optimised using gradient-based LBFGS^34^ from a central initial point:
  12 $${{\bf{C}}}^{next}=\arg \mathop{\min }\limits_{{\bf{C}}\in {[0,1]}^{d}}{\mathrm{LCB}}({\bf{C}}),$$
6. Repeat the process for the predefined number of iterations.
7. Select the best performing candidate.

### Regularisation

Under partial observability, the inverse FBA problem becomes more underdetermined, as only a subset of fluxes is available to guide the inference of the objective coefficients **c**. To improve robustness and avoid overfitting to this limited set of measurements, an L2 regularisation term is added to the loss function defined previously. The modified loss becomes:

13 $${{\mathcal{L}}}_{{\mathrm{modified}}}({\bf{C}})={\mathcal{L}}({\bf{C}})+\lambda \parallel {\bf{C}}{\parallel }_{2}^{2}$$

where ${\mathcal{L}}({\bf{C}})$ is the original data fidelity term, *λ* is a regularisation hyperparameter controlling the strength of the penalty, and $\parallel {\bf{C}}{\parallel }_{2}^{2}$ encourages smaller coefficients and smoother objectives.

This is implemented by adding a penalty term inside the optimisation loop. The regularisation parameter *λ* is selected via grid search over a predefined candidate set:

14 $$\Lambda =\{1{0}^{-6},1{0}^{-5},1{0}^{-4}\}$$

For each *λ* ∈ *Λ*, the objective coefficient vector **c** is randomly initialised and optimised for a fixed number of epochs. The best regularisation value is selected according to the final regularised loss:

15 $${\lambda }^{* }=\arg \mathop{\min }\limits_{\lambda \in \Lambda }{{\mathcal{L}}}_{{\mathrm{reg}}}^{\mathrm{fi}{\rm{n}}{\rm{a}}{\rm{l}}}(\lambda )$$

The selected λ^*^ is then used in a multi-start optimisation routine to mitigate local minima:

16 $$\mathop{\min }\limits_{{\bf{c}}}{\mathcal{L}}({\bf{c}})+{\lambda }^{* }\parallel {\bf{c}}{\parallel }_{2}^{2}$$

ensuring more stable and generalisable solutions under limited data.

### Optimisation parameters

The optimisation parameters used in both case studies can be found below, in Tables 4, 5 and 6.

**Table 4:** Optimisation stages and configurations for full observability case

Stage	Purpose	Optimiser	Initial Step Size (lr)^a^	Epochs	Other Options
Final optimisation	Train *C* from initialised value	LBFGS	1.0 × 10^−3^	50	max_iter = 20

L-BFGS adapts it via strong Wolfe line search.

^a^*lr* is an initial step-size guess.

**Table 5:** Optimisation stages and configurations for CHOmpact

Stage	Purpose	Optimiser	Initial Step Size (lr)^a^	Epochs	Other Options
Regularisation tuning	Select best lambda_reg from lambda_grid	LBFGS	1.0^a^	10	max_iter = 20, max_eval = 25, tolerance_grad = 1e-9, tolerance_change = 1e-9, history_size = 100, line_search_fn = ‘strong_wolfe’
Initialisation	Bayesian optimisation to find good starting *C*	Bayesian optimisation (Sobol sequence)	N/A	5 init pts + 10 iter	Inner eval: LBFGS (same options, lr = 1.0^a^, num_epochs = 10)
Final optimisation	Train *C* from initialised value	LBFGS	1.0^a^	50	Same LBFGS options as above

L-BFGS adapts it via strong Wolfe line search.

^a^*lr* is an initial step-size guess.

**Table 6:** Optimisation stages and configurations for iCHO2441 model

Stage	Purpose	Optimiser	Initial Step Size (lr)^a^	Epochs	Other Options
Regularisation tuning	Select best lambda_reg from lambda_grid	L-BFGS	1.0^a^	5	*λ*_reg_ ∈ {10^−6^, 10^−5^, 10^−4^}; max_iter = 10, max_eval = 25, tolerance_grad = 1e-9, tolerance_change = 1e-9, history_size = 100, line_search_fn = ‘strong_wolfe’
Initialisation	Bayesian optimisation to find a good starting *C*	Bayesian optimisation	N/A	5 init pts + 10 iter	Inner eval: L-BFGS with lr = 1.0^a^ and num_epochs = 5 (same L-BFGS options as above)
Final optimisation	Train *C* from initialised value (per start)	L-BFGS	1.0^a^	25	Same L-BFGS options as above

L-BFGS adapts it via strong Wolfe line search.

^a^*lr* is an initial step-size guess.

### Calculation of KKT Conditions for Full Observability Case

To assess whether the coefficients returned by each convex basis function correspond to optimal (local) solutions, we evaluate the KKT conditions for the full observability case. For convex objectives with affine constraints, the KKT conditions are necessary and sufficient for optimality^22,23^.

We pose the problem in ≤-form so that inequality multipliers are nonnegative:

$$\min /\max \,f({\bf{v}})\,{\mathrm{s}}.{\mathrm{t}}.\,{\bf{S}}\,{\bf{v}}={\bf{0}},\,{\bf{v}}-{{\bf{v}}}_{\max }\le {\bf{0}},\,{{\bf{v}}}_{\min }-{\bf{v}}\le {\bf{0}}.$$

Let the Lagrange multipliers be ***λ***_up_≥**0** (for ${\bf{v}}-{{\bf{v}}}_{\max }\le {\bf{0}}$), ***λ***_low_≥**0** (for ${{\bf{v}}}_{\min }-{\bf{v}}\le {\bf{0}}$) and ***μ*** (free) for the equality **S** **v** = **0**. The Lagrangian is

$${\mathcal{L}}({\bf{v}},{\lambda }_{\mathrm{up}},{{\rm{\lambda }}}_{\mathrm{low}},\mu )=f({\bf{v}})+{\lambda }_{\mathrm{up}}^{{\rm{\top }}}({\bf{v}}-{{\bf{v}}}_{\max })+{\lambda }_{\mathrm{low}}^{{\rm{\top }}}({{\bf{v}}}_{\min }-{\bf{v}})+{\mu }^{\top }({\bf{S}}{\bf{v}}).$$

The KKT conditions at a candidate solution **v**^⋆^ are:

$$\begin{array}{l}{\bf{Primalfeasibility\; :}}\,{\bf{S}}\,{{\bf{v}}}^{\star }={\bf{0}},\,{{\bf{v}}}^{\star }\le {{\bf{v}}}_{\max },\,{{\bf{v}}}^{\star }\ge {{\bf{v}}}_{\min }.\\ {\bf{Dual}}\,{\bf{feasibility\; :}}\,{{\boldsymbol{\lambda }}}_{{\mathrm{up}}}^{\star }\ge {\bf{0}},\,{{\boldsymbol{\lambda }}}_{{\mathrm{low}}}^{\star }\ge {\bf{0}}.\\ {\bf{Stationarity\; :}}\,{\mathrm{sgn}}\,\nabla f({{\bf{v}}}^{\star })+{{\bf{S}}}^{\top }{{\boldsymbol{\mu }}}^{\star }+{{\boldsymbol{\lambda }}}_{{\mathrm{up}}}^{\star }-{{\boldsymbol{\lambda }}}_{{\mathrm{low}}}^{\star }={\bf{0}},\\ {\mathrm{sgn}}=\left\{\begin{array}{c}\begin{array}{cc}+1, & \mathrm{if}\,\mathrm{minimizing}\,f,\end{array}\\ \begin{array}{cc}-1, & \mathrm{if}\,\mathrm{maximizing}\,f.\end{array}\end{array}\right.\\ {\bf{Complementary}}\,{\bf{slackness\; :}}\,{{\boldsymbol{\lambda }}}_{{\mathrm{up}}}^{\star }\circ ({{\bf{v}}}^{\star }-{{\bf{v}}}_{\max })={\bf{0}},\,{{\boldsymbol{\lambda }}}_{{\mathrm{low}}}^{\star }\circ ({{\bf{v}}}_{\min }-{{\bf{v}}}^{\star })={\bf{0}},\end{array}$$

where ∘ denotes elementwise (Hadamard) product.

In practice, Table 7 reports small residuals for each condition: ∣∣**Sv**^⋆^∣∣ (primal feasibility), the maximum bound violation, $| | sgn\,\nabla f({{\bf{v}}}^{\star })+{{\bf{S}}}^{\top }{{\boldsymbol{\mu }}}^{\star }+{{\boldsymbol{\lambda }}}_{up}^{\star }-{{\boldsymbol{\lambda }}}_{low}^{\star }| |$ (stationarity) and $\mathop{\max }\limits_{i}| {{\boldsymbol{\lambda }}}_{up,i}({{\bf{v}}}_{i}^{\star }-{{\bf{v}}}_{\max ,i})|$ and $\mathop{\max }\limits_{i}| {{\boldsymbol{\lambda }}}_{low,i}({{\bf{v}}}_{\min ,i}-{{\bf{v}}}_{i}^{\star })|$ (complementarity).

**Table 7:** KKT residuals for different convex basis functions used

Mode	Primal Feas.	Bounds Feas.	Stationarity Resid.	Comp. Slack Min	Comp. Slack Max
logsumexp	4.66 × 10^−10^	1	1.15 × 10^−5^	4.57 × 10^−6^	5.21 × 10^−6^
norm	1.70 × 10^−13^	1	8.62 × 10^−7^	1.94 × 10^−11^	2.04 × 10^−11^
sum_squares	3.52 × 10^−12^	1	1.95 × 10^−5^	1.48 × 10^−9^	1.44 × 10^−9^

### Benchmark: ObjFind*

CellTarget is benchmarked against an in-house implementation inspired by ObjFind, hereafter referred to as ObjFind*. As in the original formulation, the inverse FBA problem is recast as a single-level convex optimisation problem by explicitly encoding first-order KKT conditions of the forward FBA. However, several deliberate modifications are introduced to improve robustness under partial observability and to prevent degenerate solutions driven by excessive slack exploitation.

Let ${\bf{S}}\in {{\mathbb{R}}}^{m\times n}$ denote the stoichiometric matrix, ${\bf{v}}\in {{\mathbb{R}}}^{n}$ the intracellular flux vector and ${{\bf{v}}}^{\exp }\in {{\mathbb{R}}}^{n}$ the experimentally measured fluxes. Agreement with measurements is enforced by minimising the squared error over the subset of measured fluxes ${\mathcal{M}}$,

17 $$\mathop{\sum }\limits_{j\in {\mathcal{M}}}{({{\bf{v}}}_{j}-{{\bf{v}}}_{j}^{\exp })}^{2}.$$

Flux feasibility is imposed through steady-state constraints with tolerance *ε*_ss_ > 0 (to match CellTarget’s steady-state relaxation),

18 $$-{\varepsilon }_{ss}\le {\bf{S}}{\bf{v}}\le {\varepsilon }_{ss},$$

and lower and upper flux bounds,

19 $${{\bf{v}}}^{\min }\le {\bf{v}}\le {{\bf{v}}}^{\max }.$$

To emulate optimality of a forward FBA problem, first-order KKT conditions are partially enforced. Specifically, stationarity of the Lagrangian with respect to **v** is imposed,

20 $${\bf{C}}+{{\bf{S}}}^{\top }{{\boldsymbol{y}}}^{{\mathrm{pos}}}-{{\bf{S}}}^{\top }{{\boldsymbol{y}}}^{{\mathrm{neg}}}-{{\boldsymbol{\mu }}}^{{\mathrm{lb}}}+{{\boldsymbol{\mu }}}^{{\mathrm{ub}}}={\bf{0}},$$

where ${{\boldsymbol{y}}}^{{\mathrm{pos}}},{{\boldsymbol{y}}}^{{\mathrm{neg}}}\in {{\rm{{\mathbb{R}}}}}_{\ge 0}^{m}$ and ${{\boldsymbol{\mu }}}^{{\mathrm{lb}}},{{\boldsymbol{\mu }}}^{{\mathrm{ub}}}\in {{\rm{{\mathbb{R}}}}}_{\ge 0}^{n}$ are dual variables associated with the steady-state and flux bound constraints, respectively.

Identifiability of the cellular objective is ensured by restricting the objective coefficients **C** to a predefined candidate set ${\mathcal{O}}$ and enforcing a simplex constraint,

21 $${C}_{j}=0\,(j\,\notin \,{\mathcal{O}}),\,{C}_{j}\ge 0\,(j\in {\mathcal{O}}),\,\mathop{\sum }\limits_{j\in {\mathcal{O}}}{C}_{j}=1.$$

Complementarity conditions are not imposed explicitly. Instead, deviations from ideal KKT behaviour are discouraged through a convex regularisation term that penalises both constraint slacks and dual magnitudes,

22 $$\begin{array}{l}{{\mathcal{R}}}_{{\mathrm{KKT}}}=\,\frac{1}{m}(| | {{\boldsymbol{y}}}^{{\mathrm{pos}}}| {| }_{1}+| | {{\boldsymbol{y}}}^{{\mathrm{neg}}}| {| }_{1}+| | {\varepsilon }_{{\mathrm{ss}}}-{\bf{S}}{\bf{v}}| {| }_{1}+| | {\varepsilon }_{{\mathrm{ss}}}+{\bf{S}}{\bf{v}}| {| }_{1})\\ +\frac{1}{n}(| | {{\boldsymbol{\mu }}}^{{\rm{l}}{\rm{b}}}| {| }_{1}+| | {{\boldsymbol{\mu }}}^{{\mathrm{ub}}}| {| }_{1}+| | {\bf{v}}-{{\bf{v}}}^{\min }| {| }_{1}+| | {{\bf{v}}}^{\max }-{\bf{v}}| {| }_{1}).\end{array}$$

The resulting optimisation problem is

23 $$\mathop{\min }\limits_{{\bf{v}},{\bf{C}},{\boldsymbol{y}},{\boldsymbol{\mu }}}\,\mathop{\sum }\limits_{j\in {\mathcal{M}}}{(\bf{v}_{j}-\bf{v}_{j}^{\exp })}^{2}+{\rho }_{{\mathrm{KKT}}}\,{{\mathcal{R}}}_{{\mathrm{KKT}}},$$

which is convex. While ObjFind^13^ adopts a different formulation and assumes full observability of objective-related fluxes in *E. coli*, both approaches transform a bilevel optimisation into a single-level convex problem. The formulation presented here extends this idea to partially observed mammalian systems. For clarity, this benchmark implementation is denoted ObjFind*, where the asterisk indicates the reformulation described in this section.

## Supplementary information

Supplementary information

## Data Availability

All data supporting the findings of this study are available from the corresponding author upon request for the purposes of peer review. The data used for this work is available at https://github.com/marianaipmonteiro/CellTarget.

## Code availability

The code is available at https://github.com/marianaipmonteiro/CellTarget.
