# Supplementary material for: CellTarget: a convex optimisation approach to discover cellular objectives
Source: NPJ Syst Biol Appl. 2026 Apr 3;12:79. doi: 10.1038/s41540-026-00700-8 (PMC13230727; doi:10.1038/s41540-026-00700-8)
Supplement: Supplementary file 1 — Supplementary information [file 41540_2026_700_MOESM1_ESM.pdf]

Supplementary Information

Loss Plots

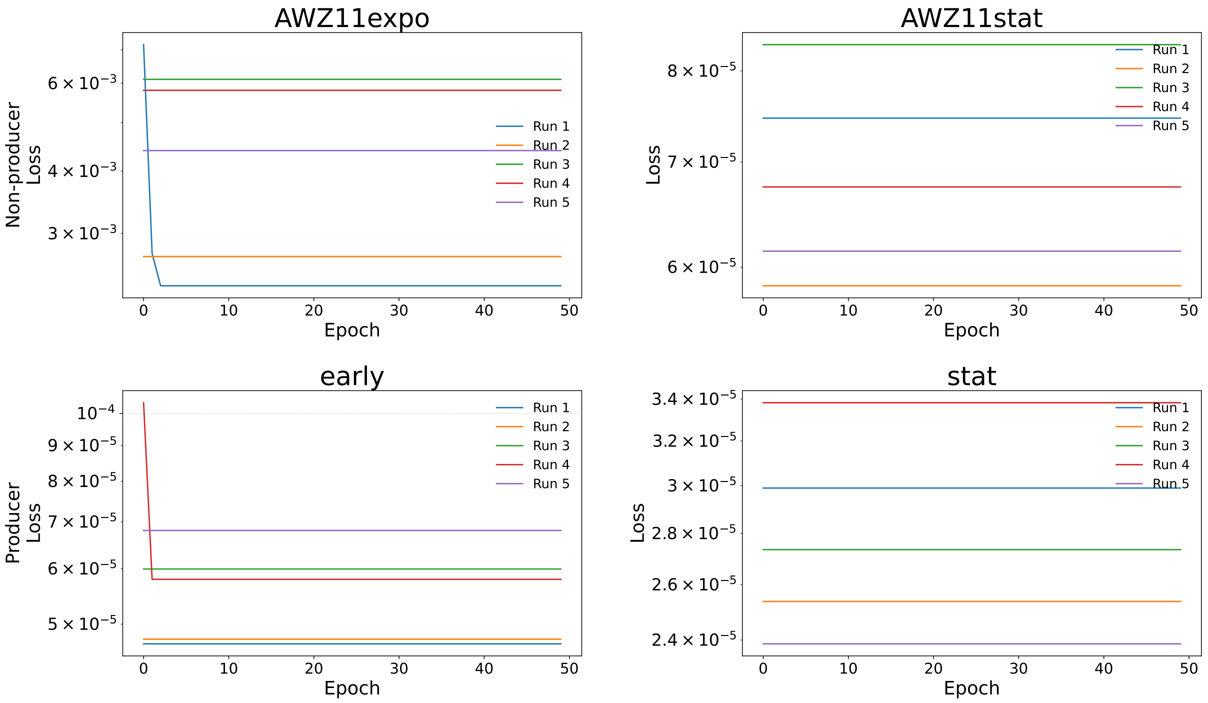

Supplementary Figure 1: **Loss for exponential growth and stationary phase, in a producer and non-producer cell lines (Cellular Objectives 'Standard').** Each subplot corresponds to one run from the multi-start initialisation procedure (Runs 1–5).

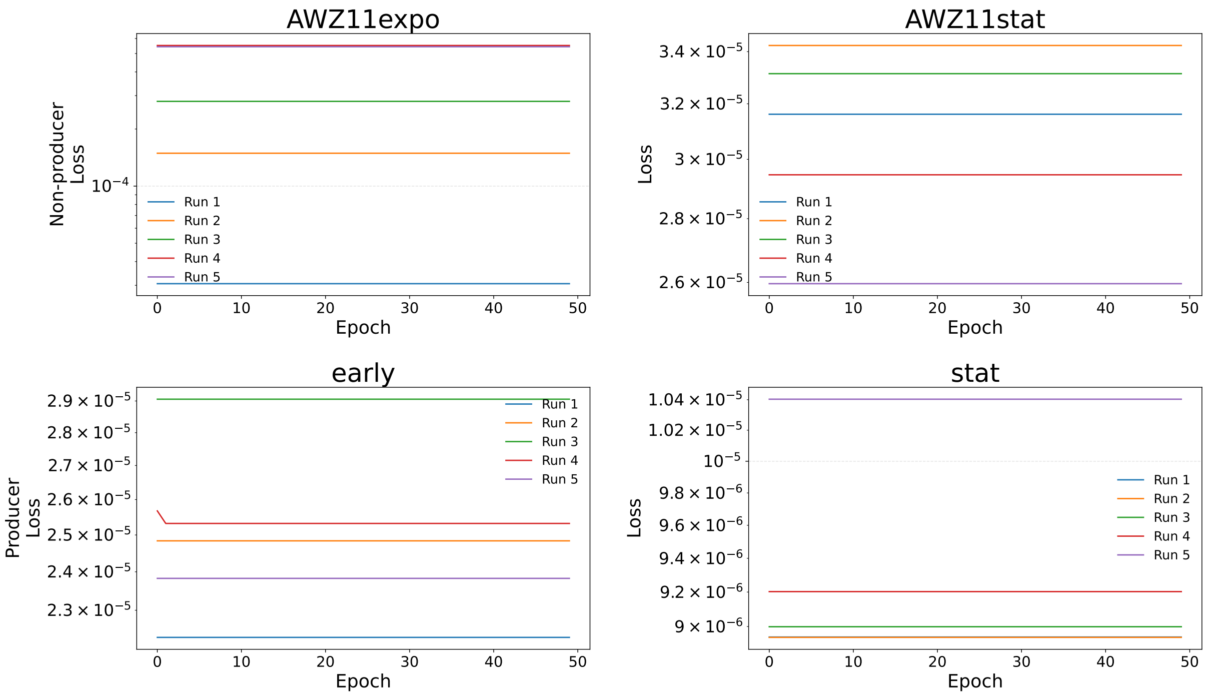

Supplementary Figure 2: **Loss for exponential growth and stationary phase, in a producer and non-producer cell lines (Cellular Objectives 'Extra').** Each subplot corresponds to one run from the multi-start initialisation procedure (Runs 1–5).

Computational Effort

Configuration	Walltime	CPU Time
standard	01:27:27	05:43:29
extra	01:00:05	02:55:04
measured	00:48:57	03:07:19
all	02:15:20	07:58:38

Supplementary Table 1: Cumulative processor time summed across all cores (CPU time) and elapsed real (clock) time from start to completion (Walltime) for the different cellular objective dimensionality conditions (for the producer cell line in exponential phase 'Early').

Configuration	Walltime	CPU Time
CHOmpact ('Standard','Early')	01:27:27	05:43:29
CHOmpact ('Extra','Early')	01:00:05	02:55:04
iCHO2441 ('Standard','Early')	48:52:58	167:59:50
iCHO2441 ('Extra','Early')	27:40:52	123:58:11

Supplementary Table 2: CPU and walltime usage for CHOmpact and GEM models under standard and extracelullar cellular objective configurations (for the producer cell line in exponential phase 'Early'). CPU time corresponds to cumulative processor time summed across five cores, while walltime denotes elapsed real time.

Effect of Model Size

The following figures show the inferred cellular objective coefficients for the CHOmpact and iCHO2441 models under for different phases and cell lines. Rows correspond to candidate reactions, and columns denote objective-set definitions (standard, extra, measured, all). Colour intensity represents the normalised magnitude of the inferred coefficient (0–1), with higher values indicating stronger contribution to the cellular objective. Blank entries indicate reactions not included in a given objective set.

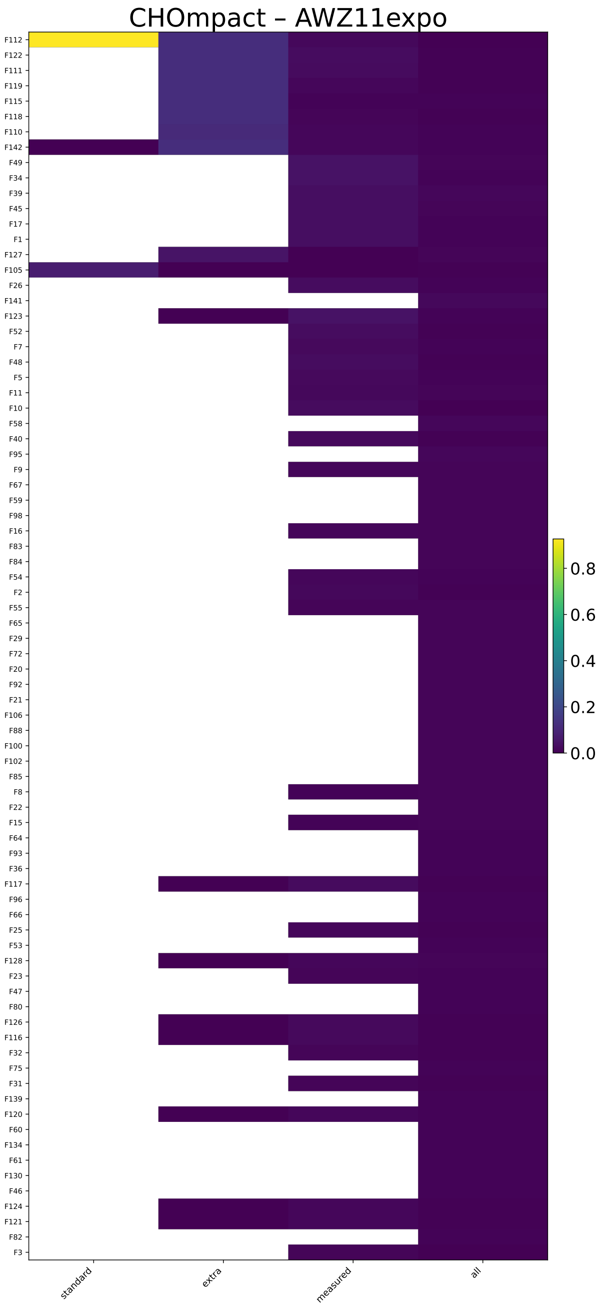

Supplementary Figure 3: **Non-producer cell line, exponential phase (CHOmpact).** The panel shows the coefficients for the AWZ11 non-producer culture during exponential growth using the CHOmpact model.

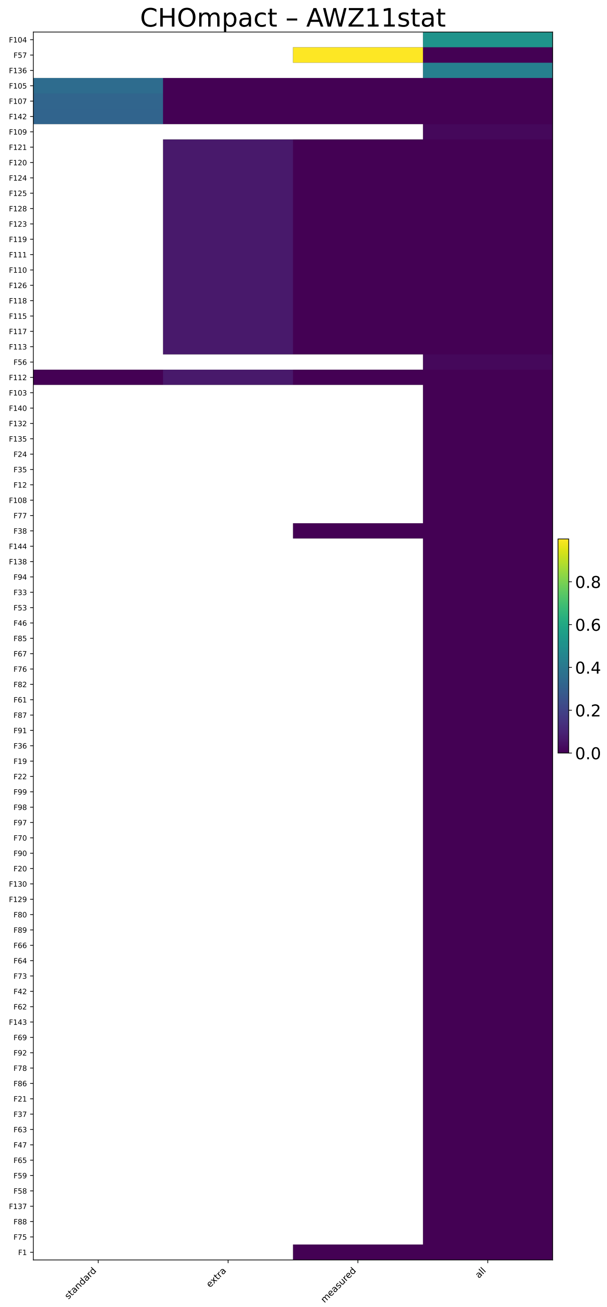

Supplementary Figure 4: **Non-producer cell line, stationary phase (CHOmpact).** The panel shows the coefficients for the AWZ11 non-producer culture during stationary phase using the CHOmpact model.

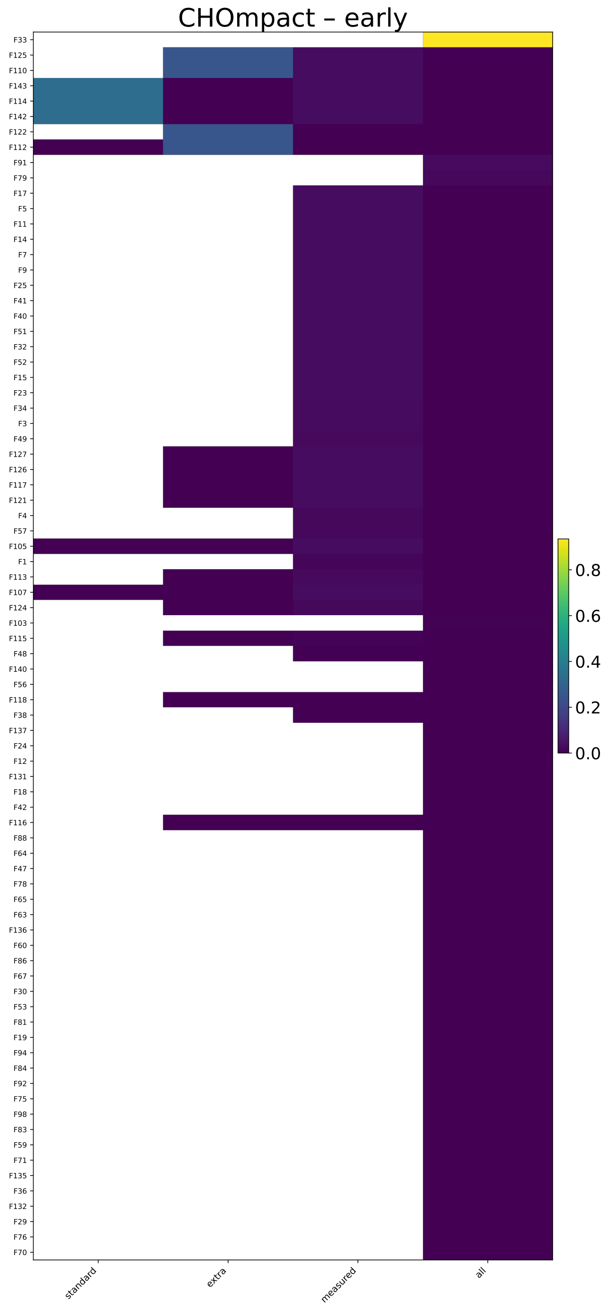

Supplementary Figure 5: **Producer cell line, exponential phase (CHOmpact).** The panel shows the coefficients for the producer culture during exponential growth using the CHOmpact model.

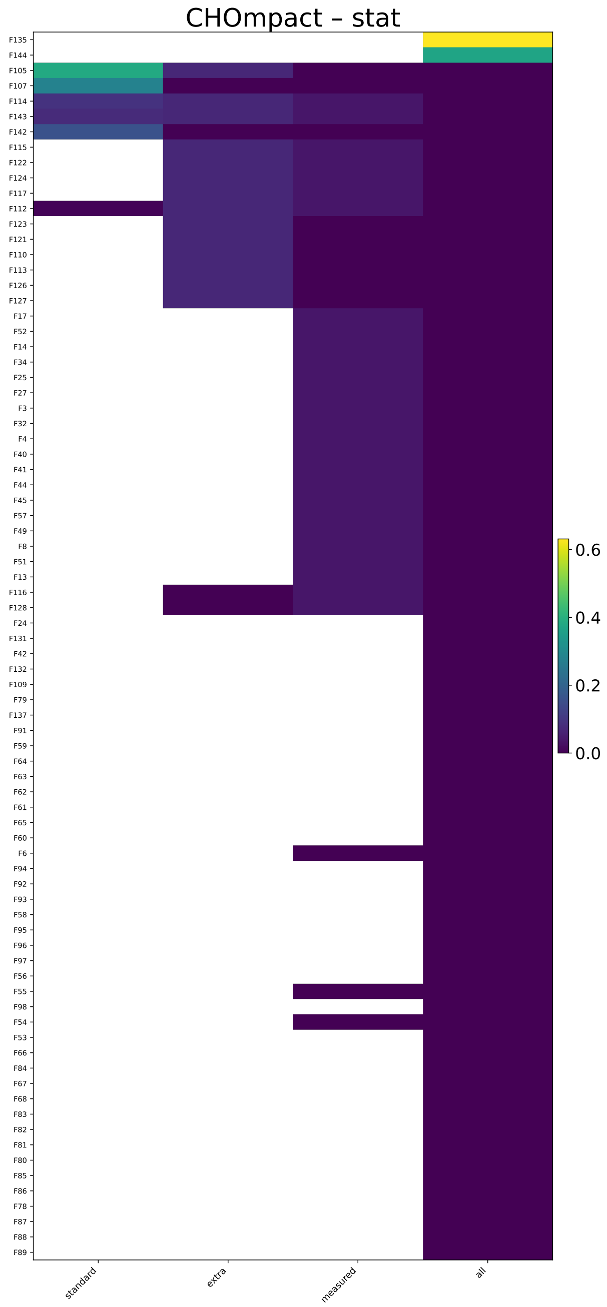

Supplementary Figure 6: **Producer cell line, stationary phase (CHOmpact).** The panel shows the coefficients for the producer culture during stationary phase using the CHOmpact model.

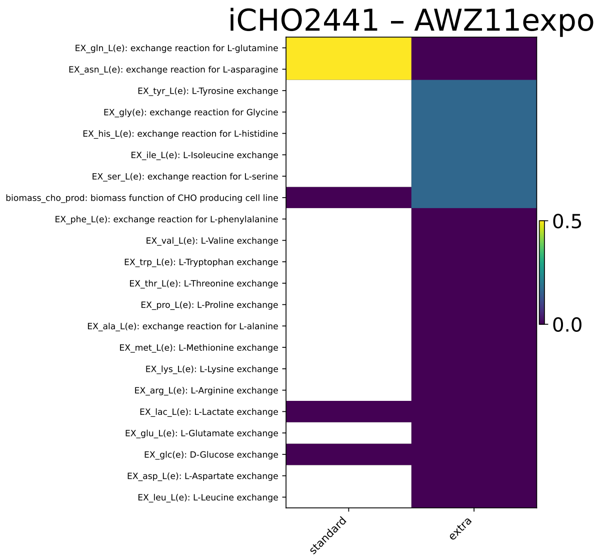

Supplementary Figure 7: **Non-producer cell line, exponential phase (iCHO2441).** The panel shows the coefficients for the AWZ11 non-producer culture during exponential growth using the iCHO2441 model.

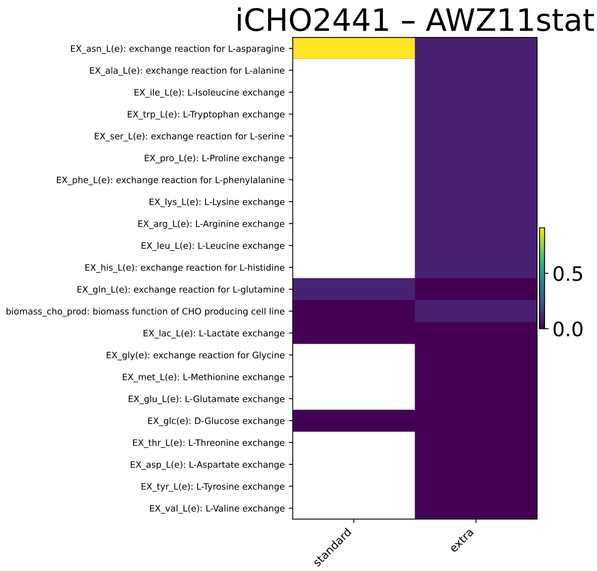

Supplementary Figure 8: **Non-producer cell line, stationary phase (iCHO2441).** The panel shows the coefficients for the AWZ11 non-producer culture during stationary phase using the iCHO2441 model.

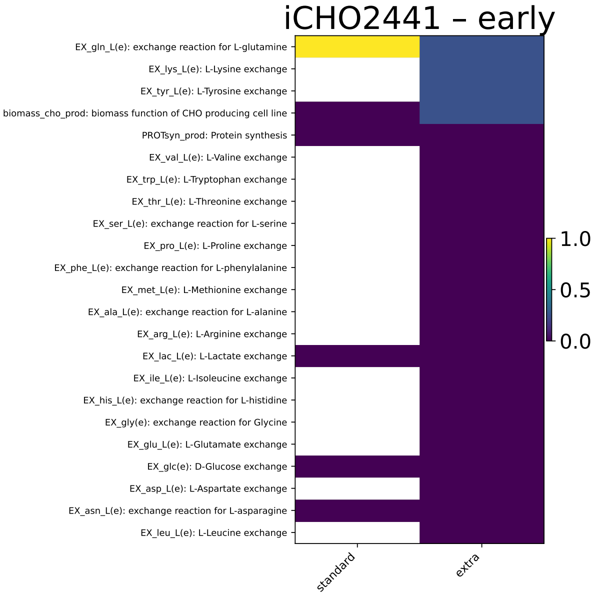

Supplementary Figure 9: **Producer cell line, exponential phase (iCHO2441).** The panel shows the coefficients for the producer culture during exponential growth using the iCHO2441 model.

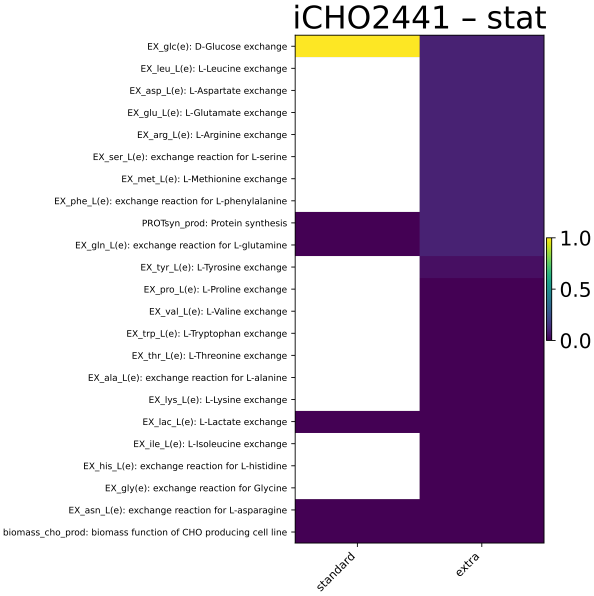

Supplementary Figure 10: **Producer cell line, stationary phase (iCHO2441).** The panel shows the coefficients for the producer culture during stationary phase using the iCHO2441 model.
